# Tracking the evolution of hospice palliative care in Canada: A comparative case study analysis of seven provinces

**DOI:** 10.1186/1472-6963-10-147

**Published:** 2010-06-01

**Authors:** Allison M Williams, Valorie A Crooks, Kyle Whitfield, Mary-Lou Kelley, Judy-Lynn Richards, Lily DeMiglio, Sarah Dykeman

**Affiliations:** 1 School of Geography and Earth Sciences, McMaster University, Hamilton, Ontario, Canada; 2 Department of Geography, Simon Fraser University, Burnaby, British Columbia, Canada; 3 Faculty of Extension, University of Alberta, Edmonton, Alberta, Canada; 4 School of Social Work and Northern Ontario School of Medicine, Lakehead University, Thunder Bay, Canada; 5 Department of Sociology and Anthropology, University of Prince Edward Island, Charlottetown, Canada

## Abstract

**Background:**

An aging population, rise in chronic illnesses, increase in life expectancy and shift towards care being provided at the community level are trends that are collectively creating an urgency to advance hospice palliative care (HPC) planning and provision in Canada. The purpose of this study was to analyze the evolution of HPC in seven provinces in Canada so as to inform such planning and provision elsewhere. We have endeavoured to undertake this research out of awareness that good future planning for health and social care, such as HPC, typically requires us to first look backwards before moving forward.

**Methods:**

To identify key policy and practice events in HPC in Canada, as well as describe facilitators of and barriers to progress, a qualitative comparative case study design was used. Specifically, the evolution and development of HCP in 7 strategically selected provinces is compared. After choosing the case study provinces, the grey literature was searched to create a preliminary timeline for each that described the evolution of HPC beginning in 1970. Key informants (*n *= 42) were then interviewed to verify the content of each provincial timeline and to discuss barriers and facilitators to the development of HPC. Upon completion of the primary data collection, a face-to-face meeting of the research team was then held so as to conduct a comparative study analysis that focused on provincial commonalities and differences.

**Results:**

Findings point to the fact that HPC continues to remain at the margins of the health care system. The development of HPC has encountered structural inheritances that have both sped up progress as well as slowed it down. These structural inheritances are: (1) foundational health policies (e.g., the Canada Health Act); (2) service structures and planning (e.g., the dominance of urban-focused initiatives); and (3) health system decisions (e.g., regionalization). As a response to these inheritances, circumventions of the established system of care were taken, often out of necessity. Three kinds of circumventions were identified from the data: (1) interventions to shift the system (e.g., the role of advocacy); (2) service innovations (e.g., educational initiatives); and (3) new alternative structures (e.g., the establishment of independent hospice organizations). Overall, the evolution of HPC across the case study provinces has been markedly slow, but steady and continuous.

**Conclusions:**

HPC in Canada remains at the margins of the health care system. Its integration into the primary health care system may ensure dedicated and ongoing funding, enhanced access, quality and service responsiveness. Though demographics are expected to influence HPC demand in Canada, our study confirms that concerned citizens, advocacy organizations and local champions will continue to be the agents of change that make the necessary and lasting impacts on HPC in Canada.

## Background

In Canada, as with many other developed nations, demographic trends confirm an increase in the elderly population as the generation affectionately known as the 'baby boomers' reaches retirement age, the fertility rate decreases, and the average life expectancy increases due to advances in health care [1,2]. While Canadians are indeed living longer, many are doing so in poor health as levels of chronic disease are on the rise, accounting for nearly 70% of all deaths in Canada [3]. This trend towards growth in the aging population and the rise in chronic illness, particularly in the later stages of life, amplifies demand for hospice palliative care (HPC) services. Additionally, more Canadians are voicing a desire to die in their homes [4-6], which is coinciding with a shift of end-of-life care from hospitals and acute care facilities into the community [7,8]. Given that, there has been a shift in Canadians' place of death out of hospitals and into community settings, particularly after hospital deaths peaked in 1994 [9,10]. There is thus a pressing need to address not only the anticipated growth in demand for HPC, but specifically forms of care that can support home death.

Canada is recognized as an international leader in the provision of HPC care [3]. In 1975, Canadian physician Dr. Balfour Mount coined the term 'palliative care' as it is used in the modern context [11]. The term has since evolved to include the philosophy of hospice care and come to receive the HPC designation. In Canada, HPC began in the 1970s, at the same time that cancer treatment centres identified and prioritized pain and symptom management [12,13]. In 1991, a national body was established, then called the Canadian Palliative Care Association and presently known as the Canadian Hospice Palliative Care Association (CHPCA) [3]. Now, almost 40 years later, a national Senate Committee report asserts that every Canadian is entitled to " die in relative comfort, as free as possible from physical, emotional, psychosocial, and spiritual distress [with] access [to] skilled, compassionate, and respectful care" [14]. Despite this assertion, the provision of HPC in Canada remains a work in progress.

Driven both by an acknowledgement that many dying Canadians and their family caregivers still do not receive adequate HPC, and that demand for such services is only expected to grow, a commitment to enhance HPC across the country exists [14]. In order to undertake such enhancement, it is essential to understand how HPC in Canada has evolved since its early days to become the kind of care that it is at present so as to best inform future decision-making. This is, however, no easy task as HPC in Canada can be described as a 'patchwork-quilt' of services and programs provided inconsistently across both place and time [3].

To understand the structures and process that have served as facilitators and barriers in shaping the development of Canadian HCP, a systematic analysis was undertaken. In this paper, we report on the evolution of HPC in seven Canadian provinces selected to represent Canada's diversity. Relying on a thematic analysis of key informant interviews (n = 42) and grey literature, we aim to examine our key finding herein: that there are structural inheritances in place that have both facilitated and slowed down the advancement of HPC in Canada that have sometimes led to often innovative circumventions being put in place. We believe that the framing of this analysis around 'inheritances and circumventions' provides a conceptual contribution that is unique to the health services literature, while our findings provide much needed systematically-gathered evidence about the past and present state of HPC provision required for informing future decision-making.

## Methods

The overall objective of this study was to determine the evolution of HPC in targeted Canadian provinces by identifying key policy and practice events along with barriers and facilitators to progress. To achieve this we employed a qualitative comparative case study design. The case study methodology is ideal to use in studies posing 'how' (e.g., *how *has HPC evolved in different Canadian provinces?) and 'why' (e.g., *why *has HPC evolved in the way it has?) questions about a single ongoing event where context is highly relevant [15]. We employed the case study methodology in a comparative manner because of the highly localized nature of the context of HPC in each province (e.g., political will, health service structure). Each of the seven provinces, therefore, was treated as a distinct case. Furthermore, qualitative approaches are appropriate to use in exploratory studies such as this one, especially when little is known about the topics being examined and testable hypotheses cannot be defined [16,17]. The specific methods of data collection and the analytic techniques employed have been informed by the overall qualitative comparative case study design.

This study took place in its entirety from the summer of 2006 to the summer of 2009, after first receiving ethics approval from each of the participating universities. The first step in the research process was to identify the individual case study provinces. Seven provinces were purposefully selected that together represent Canada's linguistic, cultural, political, regional, and social diversity, namely (from west to east): British Columbia, Alberta, Saskatchewan, Manitoba, Ontario, Quebec, and Prince Edward Island (see Table 1 for an overview of province characteristics). All provinces except for Quebec are mainly English speaking. Members of the investigative team each took responsibility for different cases to enable immersion in a province's context.

**Table 1:** Study Province Characteristics

Province	Total Population (2009/2006)- persons (thousands)*		Median Age (2008)*	Percentage of population over the age of 65 (2006)*	Sex ratio (number of males for 100 females) (2006) *	Regionalization- Established/changed (year) **
					
	2009	2006				
British Columbia	4,455.2	4,243.6	40.5	14.6	95.9	1997/2001

Alberta	3,687.7	3,421.3	35.7	10.7	100.2	1994/2003

Saskatchewan	1,030.1	992.1	37.9	15.4	96.4	1992/2001-2002

Manitoba	1,222.0	1,184.0	37.8	14.1	96.3	1997-1998/2002

Ontario	13,069.2	12,665.3	39.0	13.6	95.2	2005

Quebec	7,828.9	7,687.1	41.0	14.3	95.6	1989-1992/2003

Prince Edward Island	141.0	137.9	41.3	14.9	93.4	1993-1994/2005

**Canada**	33,739.9	32,576.1	39.4	13.7	95.9	

*Statistics Canada-http://www.statcan.ca

**As cited in Marchildon (2006).

Following identification of the case study provinces, review of the relevant grey literature was undertaken to identify preliminary HPC evolution timelines for each provincial jurisdiction. The review of this literature focused on gathering relevant government policy documents and reports from HPC organizations. Because, by its very nature, grey literature is comprised of materials not typically identifiable through standard bibliographic searches [18], team members avoided using the typical literature search engines and instead hand searched websites of key provincial organizations and ministries and also contacted HPC researchers and/or clinicians to obtain documents otherwise unavailable. When the point where no new HPC documents relevant to the study were being identified was reached, team members then focused on synthesizing the content of the grey literature using five guiding categories: (1) milestones (e.g., when key policy and practice events occurred); (2) context (e.g., names and mandates of agencies and departments involved); (3) concerns (e.g., established HPC foci and their changes over time); (4) policies and practices (e.g., relevant health policies); and (5) outcomes (e.g., the impacts of events, policies, and practices over time). These summative categories were derived by adapting Blaikie and Soussan's policy process analysis matrix [19]. Data relevant to each of these five categories were extracted from the sources and stored in synthesis spreadsheets. The data were also used to generate preliminary HPC evolution timelines for each of the case study provinces.

Upon completion of the grey literature review and synthesis of preliminary timelines, interviews with key informants were conducted to assist with finalizing the HPC timelines for each case study province and gathering further contextual information relevant to the overall objective. Key informants were identified in one of three ways, either by: (1) identifying names mentioned consistently in the grey literature documents; (2) asking established HPC researchers and decision-makers known by the research team to suggest potential interviewees; or (3) listening for people and organization names raised during key informant interviews. We specifically interviewed key informants with expertise in HPC advocacy, administration, decision-making, and policy-making.

Key informant interviews were conducted by telephone. In advance of the interview, key informants were sent consent information and the preliminary HPC evolution timeline. Interviews were semi-structured and probed: critical turning points in HPC; social and political forces that have impacted the evolution of HPC; and key challenges and successes that have informed HPC policy and practice. Key informants were also asked to augment or confirm the HPC preliminary timeline. This proved to be a particularly useful way to open up a conversation about particular HPC events and milestones. Key informants were asked to specifically discuss their respective provinces during the interview, though comparative references to other provinces or reference to national events and milestones were permitted. In total, 42 key informant interviews were completed, seven in each of British Columbia, Alberta, Manitoba, and Ontario, six in Saskatchewan, and four in each of Prince Edward Island and Quebec. All interviews were digitally recorded and were transcribed verbatim upon completion.

To effectuate the *comparative *case study analysis, a face-to-face team meeting was held with the intent to collaboratively and thematically analyze the data (i.e., key informant transcripts, grey literature syntheses, and finalized HPC evolution timelines), compiled across the seven case studies. We focused on key commonalities and differences across the provinces using thematic analysis. Thematic analysis involves categorizing data according to units that can be identified from patterns within the dataset, known as 'themes', which are then compared to the existing literature and study objectives [20]. The analysis meeting started with the investigators presenting 'case study reports' for each of the seven provinces studied. The team then moved to comparatively identify apparent similarities and differences across the cases, expressed using cross-cutting themes. The next step was to identify an explanatory framework that helped to further explain the cross-cutting themes through asking such questions as: *why *did event X happen in this province alone; *how *did all provinces come to offer Y service in similar ways; and *what *were the common and different impacts of federal initiative Z across the provinces? Through this inquiry-based process, a framework of inheritances and circumventions was identified. We found this to be a meaningful way to explain the comparative findings of the case studies and to identify the themes that contribute to the framework by providing relevant examples from the different provinces.

## Results

**Figure 1:**
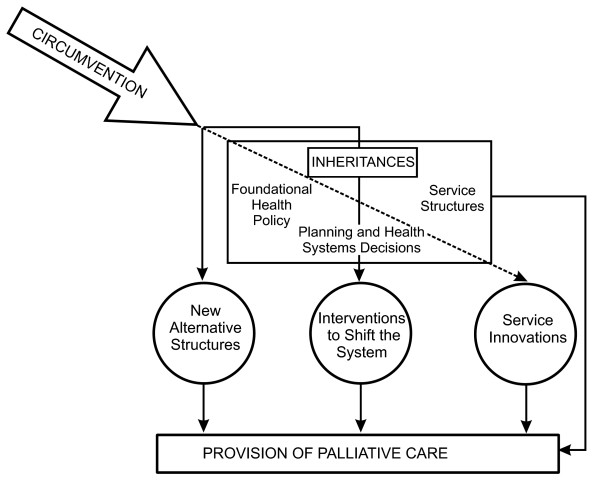
**A conceptual model of the evolution of hospice palliative care in Canada**. The path through which HPC is provided in Canada is depicted in this figure. Due to existing inheritances, which include: (1) foundational health policies; (2) service structures; and (3) planning and health systems decisions, provision of HPC services is surmised to flow in four separate ways. The first route is the most direct, which involves providing HPC in strict adherence to the inheritances. Indirect paths are illustrated through the three forms of circumventions: (1) new alternative structures; (2) interventions to shift the system; and (3) service innovations. As shown, new alternative structures completely bypass the inheritances, and as such do not have to adhere to the policies outlined by the inheritances. Interventions to shift the system work within the confines of the system to promote the need to change or amend the inheritances. Finally, service innovations can be understood to use the inheritances as a foundation upon which to build new HPC approaches and models

Recognizing that HPC has and continues to remain at the margins of the health care system, our inheritances and circumventions framework assumes that there are structural inheritances in place that have both facilitated and slowed the advancement of HPC in Canada. When these inheritances slowed advancement of palliative care, they often stimulated actions intended as circumventions, i.e. intended to 'go around' the established system of care. Figure 1 visually depicts our findings, illustrating this framework and establishing the interrelationships among the inheritances and circumventions identified in the data. In the remainder of this paper we expand on this framework and present the themes relevant to its central components.

### i) Inheritances

Although not depicted in the figure, due to the all encompassing nature of culture, Canadians have largely inherited a culture of health care delivery characterized as both highly curative and bio-medical in nature and, thereby, more interested in healing the bio-physical body rather than attending to the psycho-social and spiritual elements of the dying being [21]. Further, this care system feeds off of and into a larger death-denying culture, where the experience of dying is not yet comfortably managed by the Canadian population at large [22]. Similar to the culture of health care delivery, this death-denying culture operates as an underlying inheritance. Above and beyond these basic inheritances intrinsic to the Canadian experience, key informants in this study made reoccurring reference to the scope and impact of specific national and provincial policies and practices, thus demonstrating an awareness of the importance of these founding inheritances for the development of HPC. Many of these system structures existed before HPC was formally developed in Canada and thus have intrinsically shaped the development of this form of care. Inheritances most often referred to in our data were: (1) foundational health policies such as the Canada Health Act (CHA), together with national and provincial funding structures; (2) service structures and planning processes, such as the largely urban focused health system, and the speed at which health service progress takes place; and (3) health system decisions such as regionalization. Each of these is discussed in detail in this subsection.

### 1) Foundational Health Policy

To understand the basis of health service delivery in Canada one must review how Canadian health care is governed, funded and delivered. To address governance, we turn to the Medical Care Insurance Act in 1966; it is the basic legislation intended to provide national health insurance and is designed to ensure that all Canadians have equitable access to physicians and hospital care. Following the Medical Care Insurance Act, the Canada Health Act (CHA) and the Canada Transfer Act (CTA) were enacted and have since been foundational tenants to Medicare (i.e., the publicly- funded health care system) in Canada. These Acts have been inherited over time, providing the structural legacy of healthcare and its delivery across the country.

### 1a) Canadian Health Act

The CHA is perhaps the foremost inheritance affecting the current delivery of HPC, if not all forms of health care in Canada. The CHA, enacted in 1984, advocates five principles of health care in Canada: accessibility, universality, portability, comprehensiveness and public administration [23]. This legislation conceptualized health in terms of social justice and aspired to ensure equitable access to achieve health for all so as to "protect, promote and restore the physical and mental well-being of residents of Canada and to facilitate reasonable access to health services without financial or other barriers" [23]. The CHA legislates a system of funding transfers for health care from the federal-level to the provinces, which ultimately funds community-level primary, secondary, and tertiary care services [13]. With the exception of veterans, First Nations people living on reserves, Inuit, members of the armed forces, Royal Canadian Mounted Police, and inmates of federal penitentiaries [13], the CHA outlines that the planning, financing and delivery of health care services is the responsibility of individual provinces and territories, with funding assistance being provided from the federal government [23].

The CHA is provincially regulated. As a result, there is not a standardized system of care across the provinces. There are variations in funding allotments, administration, and coverage of services among the provinces and territories [13,24]. This point is of great significance because while Canada is often referred to as having 'national health care', there is, in fact, a different system of health care provided in each province and territory. Therefore, differences in access to, and quality of, care exist across provinces and territories. There are also limitations to coverage depending on where care is delivered [23]. For example, there are discrepancies in coverage for home and community care depending on their recognition as a professionally-delivered service (e.g., delivered by a physician in a hospital versus delivered by a home care worker in the community) [23]. This is often considered an out-dated outcome of the original 1983 definition of "medically necessary care" as being what the public system provides and a sign that the CHA has not kept pace with the way services are now delivered [25].

The funding and delivery of HPC care often falls foremost within core public services: hospital care; long-term care and; homecare [26]. This creates a challenge as there is little *dedicated *funding for HPC as it falls within the funding envelope of other services regulated by the CHA. This has resulted in an uneven prioritization of palliative care across regions and provinces due, in part, to a lack of direction from the CHA, along with lack of standardization for, and thus inequity in, service provision. As Marchildon explains: "There is no national policy on palliative care in Canada. Instead, there are national guidelines developed by community-based palliative care organizations operating at arm's length from the government" [13]. As a key informant explained, this situation makes it difficult to designate certain services for HPC: "*when we come then to terminal illness, end-of-life, palliative, it gets very murky as to whether there should be more dedicated beds and facilities and locations, or not*." Generally, key informants identified the lack of funding in the public system for HPC as a significant limitation of current Canadian health policy and services.

### 1b) Health Funding Structures

Canada's health insurance program, known as Medicare, is comprised of 13 health insurance plans -- one from each of the ten provinces and three territories [27]. The federal government contributes funding to each province and territory to assist with the administration and delivery of health care services through the annual Canada Health Transfer (CHT), as legislated by the CHA. The CHT is based on a formula that ensures each province and territory receives the same per capita amount of health care funding. In order to receive this funding from the federal government, each province and territory must follow and adhere to the principles of the CHA. The majority of health care dollars are generated through taxation. Since the CHA does not specify funding for HPC services, provincial and/or non-governmental funding must be allocated to provide necessary HPC services.

Above and beyond the CHT, the federal government invested $800 million in the Primary Health Care Transition Fund (PHCTF) from 2004 to 2006 to enhance primary health care services. The purpose of this funding was to support provinces and territories in formulating better ways to deliver primary health care through new approaches and innovations [28]. Funding was allotted on a per capita basis, with the exception of the territories and Prince Edward Island; they received additional funding to compensate for their smaller populations. A seminal rural HPC educational initiative, Pallium, was funded from the CHT.

Funding from governmental and non-governmental sources have enabled the establishment and continued development of programs and resources for HPC. Key informants, however, told us that these strategic funds, whether government or non- government, are often reactive, rather than proactive. In other words, while the allotment of funds based on identified HPC needs is positive, it can lead to provincial and regional inequities in the access to and quality of care and leave individual communities responsible for filling the gaps. One example of this is Manitoba's Jocelyn House, which was Canada's first free-standing hospice built in 1985 without provincial funding. Related to this, a key informant from Alberta described how volunteer groups help to raise funds for community hospices to supplement the contribution of the publicly-funded regional health authorities (RHA):

The health authorities cannot raise capital funds. That's not in their mandate. They can't do fundraising for capital, so they're dependant on the communities right now to build hospices. And then...so what you're getting is this push-pull with hospices societies saying "If we're going to fundraise, we're going to have the hospice the way we want it.

The relationship between HPC volunteer societies and RHAs can sometimes create friction in terms of funding priorities. However, this situation is also unavoidable due to the funding limitations imposed by the CHA and CHT on which HPC services and sites can be funded with public monies.

### 2) Service Structures and Planning

Key informants observed that deficiencies in health service structures and planning have negatively impacted the delivery and overall advancement of Canadian HPC. As with those health policies discussed, many of these structural features are slow to change and were in place before the rise of the HPC movement. Placement of palliative care services within the formal health care system thus positions them as inheritances.

### 2a) Pace of Urban-Centric Health Service Progress

The establishment of community-based HPC programs is described as an ongoing process which can be expected to take a single committed community between six and ten years [29]. Indeed, our data reveals that the history and trajectory of HPC in Canada follows a similar slow pace witnessed in rural Canada [29]. For example, while some provincial palliative care associations were being established as early as the 1980s, a national guideline for HPC was not drafted until 2002, more than 20 years later.

Throughout its evolution, HPC in Canada has been decidedly urban-centric, as with most other forms of health services. Although this urban-focused care model may best meet the needs of the majority of the population since 80% of Canadians live in urban areas [30], it creates serious deficiencies in care provision in Canada's vast rural and remote expanses [29,31]. For example, a study of home care programs in Ontario uncovered a geographical and cultural bias as these programs were developed in the province's highly populated south and later superimposed on northern/rural regions [32]. In doing so, geographical and cultural differences were not considered and, as a result, programs implemented were incompatible with the needs of northern residents. Our key informants revealed that the urban-focused design of HPC services is being 'rolled out' in very much the same way in rural jurisdictions across Canada.

The legacy of urban-centric HPC care infrastructure has received varied responses across provinces and regions. For example, in Ontario, rural and remote citizens have been vocal about the fact that the limited access to, and quality of, HPC care is due to a lack of relevant education and training opportunities in non-urban areas [31]. For example, the lack of health human resources is more pronounced in rural areas than in urban ones, resulting in family doctors having to provide more specialist services to their patients when compared to their urban counterparts [33]. Recognizing that family caregivers provide the vast majority of all HPC care across geographies and settings in Canada [34], rural family caregivers often have comparatively fewer formal services to work with when compared to their urban counterparts. As one key informant observed:

If you compare rural to urban then I would say... Fairly poor with pockets of some good programming. It's quite concerning about the lack of supportive caring in rural communities. I think we have left a lot more on the families' backs in the rural community. There are some good programs across the country but I don't think we can say that we are doing that well at least I wouldn't be satisfied with where we are at.

The limited progress of developing HPC services to meet community needs underlines the slow pace at which health services progress. This is particularly true in rural and remote areas of the country.

### 3) Health Systems Decisions

A number of intentional health systems design decisions, often occurring at the provincial level, have greatly impacted the availability of HPC services in Canadian communities. The two most frequently discussed issues by the key informants were regionalization and the unclear definition of 'core services' in HPC.

### 3a) Increasing Regionalization

The regionalization of health care across Canada generally began in the 1990s with an intention to enhance health care services and improve the continuity of care [13]. The process of regionalizing health care was most often undertaken by provincial organizations responsible for allocating federal transfer monies. Broadly, the regionalization of health service delivery in Canada has involved devolving decision-making power to intra-provincial regions and communities and away from provincial jurisdictions with the goal of improving responsiveness to local needs and circumstances.

Many key informants discussed health care regionalization as a double-edged sword in relation to HPC. The process of devolving decision-making responsibilities to more regionalized jurisdictions -- namely RHAs -- has provided the opportunity for decision-makers to implement changes in response to localized HPC needs. For example, in the case of Saskatchewan the establishment of 32 RHAs in 1992 brought HPC to the forefront of health services discourse in the province. Further, the establishment of RHAs has also enabled greater collaboration between rural and urban communities, fostering partnerships which were said to enhance HPC planning and the creation of regionally-based care networks that cater to communities' specific and sometimes unique needs. Some regional HPC programs also set the standard for care in other regions, thereby acting as a driver for best practice. In contrast, regionalization has more commonly resulted in a fragmented delivery of HPC services, particularly when funding is inadequate. For example, the redistribution of RHA boundaries in Alberta in 2003 led to the restructuring of programs, clinician job loss, and the closure of the Chinook Hospice.

In regionalized models of health care each decision-making body is autonomous [35,36]. Because of this, regionalization in Canada has meant that HPC services and modes of delivery are not homogenous across regions even within the same province or territory, let alone nationally. As a key informant from Alberta pointed out, one RHA might view HPC as: *"[one] little local hospital...[with] a room that is very beautifully decorated...to provide palliative care" while another RHA might view it to be: "a coordinated regional program with outreach that includes training [and] education of primary health care workers*." This lack of shared understanding of what constitutes HPC across RHAs can also be observed in discrepancies in funding allocations and service prioritization. Regionalization can result in differences in the quantity, quality, and type of HPC services being offered within a province/territory, which is what the key informants reported to be happening across the country.

### 3b) Identification of Core Services

A number of key informants pointed out that when health services are formally identified as 'core services' there is more accountability involved in the allocation and spending of funding. For example, one key informant discussed the accountability measures that ensued when HPC was mandated as a core service through the creation of health districts in Saskatchewan in 1992: "*Now that was significant because it brought palliative care to the forefront, and people had to use [the funds] for palliative care. They could not use it anywhere else in the funding process*." In contrast, when there is no designation of core service attached to a particular health service, there is little to no accountability for RHAs to allocate funding to these services. As one key informant explained, this leads to decision-making challenges:

...there's a collective voice for [HPC in] a [regional] health authority -- but within some of them... they placed End-of-Life Care under Seniors and Spiritual Care and gave it to that Director. They're set with limited powers. They have some jurisdiction for residential [HPC], but they don't have any jurisdiction over acute care issues in palliative care. And so you get like 'Who's on first? Where do I go to address an issue'?

When HPC is not identified as a core service it becomes embedded within existing funding envelopes, thus limiting the possibility for service provision and service diversification across health care settings.

The data reveals that the development and evolution of HPC across Canada has had to manage a set of inheritances that, over time, have dictated the ways in which health services are funded, allocated, planned and delivered. The obstacles and challenges imposed by these inheritances have necessitated the development of ways to bypass and/or overcome pre-existing organizational structures, which are referred to here as circumventions.

### ii) Circumventions

The obstacles and challenges imposed by the inheritances reported on above have necessitated the development of ways to bypass or overcome pre-existing organizational structures, processes referred to here as circumventions. A circumvention is understood to be a type of action undertaken by an individual or group of individuals to overcome the limits of inherited systems or structures. Three forms of circumventions were identified by our key informants: (1) interventions and initiatives to shift the system; (2) service innovations; and (3) new alternative structures. Key national HPC events are summarized in Table 2.

**Table 2:** Key national HPC events

1991	-Canadian Palliative Care Association deemed a national charitable organization
1995	- Release of the Senate Committee report *Of Life and Death*

2000	- Release of the Subcommittee of the Standing Senate Committee on Social Affairs, Science and Technology report *Quality End of Life Care: The Right of Every Canadian *(commonly referred to as Carstairs' report)

2001	- Secretariat on Palliative and End-of-Life Care established; Senator Carstairs appointed as federal Minister with Special Responsibility for Palliative Care

	- Canadian Palliative Care Association changes to Canadian Hospice Palliative Care Association

2002	- Release of the Kirby report *The Health of Canadians- The Federal Role*
	- Release of the Romanow report *Commission on the Future of Health Care in Canada*

2004	- Primary Care Health Transition Fund begins & funds Pallium project (rural palliative care)
	- Compassionate Care Benefit enacted

2006	- Primary Health Care Transition Fund ends (Pallium enters evaluation phase)

2007	- Secretariat on Palliative and End-of-Life Care disbanded

### 1) Interventions and Initiatives to Shift the System

By tackling the inherited organizational structures, interventions and initiatives to shift the system aim to induce change in both system infrastructure and legislation across a number of levels - federal, provincial and local. Three such interventions and initiatives specific to HPC were identified by key informants, the development of: (1) specific commissions and reports on HPC care; (2) funded policy initiatives, such as the Compassionate Care Benefit and HPC drug programs; and (3) advocacy on behalf of concerned citizens, organizations and individual local champions.

### 1a) Commissions and Reports

The lack of formal HPC health care policy received national attention in 1995 as a result of a Supreme Court case. Sue Rodriguez argued that her rights, under the Canadian Charter of Rights and Freedoms were infringed upon when her wish for assisted suicide was denied. Though the Supreme Court upheld the law against assisted suicide, the marginal majority decision prompted a review of the issue by the Special Senate Committee on Euthanasia and Assisted Suicide in a report entitled *Of Life and Death *[37]. Although little immediate action developed for HPC in Canada as a result of this report, a second review was undertaken to specifically addressed directions for action [14]. As a result, the federal Standing Senate Committee on Social Affairs, Science and Technology produced a report in 2000 entitled *Quality End-of-Life Care: The Right to Every Canadian *[14]. This report provided the first national policy initiative to shift the delivery of HPC in Canada. The Honourable Sharon Carstairs, the Senator in charge of this initiative, prepared a list of recommendations designed to develop HPC, which included a call for: the development of a national HPC strategy; enhancing national discourse around HPC funding initiatives and; providing income security for informal caregivers of dying persons [14]. This report acted as a major catalyst for increased attention to HPC issues. For example, it put pressure on the federal government to devolve additional resources to provinces/territories in order to better improve HPC service provision. Thus, coinciding with increased governmental attention on HPC in the early 2000s, publications such as the Senate reports (1995, 2000), and the national reviews of the Canadian health care system provided by Kirby (2002) and Romanow (2002), sustained the momentum in advocating the community's role in advancing Canadian HPC.

### 1b) Funded Policy Initiatives

In addition to assisting with advocacy, various federal commissions and reports have led to funded HPC initiatives designed to shift the system. One national policy initiative funded through Health Canada was the establishment of the Secretariat on Palliative and End-of-Life Care, which ran from 2001 until 2007. An annual budget between $1 to 1.5 million dollars was provided to create a national strategy for HPC. Specific HPC working groups run through the Secretariat included: research, surveillance, public information and awareness, professional education and best practices and quality care. Unfortunately, the Secretariat was disbanded in 2007, and has not yet produced a formal synthesis report since entering its evaluation and analysis stage [38]. There was a final report of the HPC Secretariat that summarizes the outcomes of each of the standing committees.

The implementation of the Compassionate Care Benefit was the result of the work of the Secretariat. This federal Benefit offers eligible informal caregivers with a six week paid leave from work to provide compassionate care to a dying family member who is at risk of dying within six months [39]. The program is administered through a contributory benefits scheme known as Employment Insurance administered by a federal ministry (Service Canada). The establishment of the Compassionate Care Benefit program marks a major step forward for the Canadian government in formally developing HPC policy that recognizes the importance of the health and well-being of family caregivers and the enormous contribution associated with providing emotional, physical, spiritual and mental care to HPC patients so they can die with dignity [40,41].

The targeted HPC monies devolved from the Primary Health Care Transition Fund in 2004-2006 were used differently across provinces/territories as defined by the ministries of health. For example, in Ontario, the Ministry of Health and Long Term Care pledged $115.5 million over a three year period to the End-of-Life Care Strategy which included enhanced funding for home care services for palliative care clients, residential hospices, hospice volunteer visiting programs and the implementation of HPC Networks in each of the province's 14 Local Health Integration Networks [42]. HPC Networks are bodies of stakeholders who promote the provision of quality HPC in their individual region [43].

Further, provincial governments have shown evidence of recognizing opportunities for funding frameworks and initiatives designed to shift the system. For example, in 1992, and continuing on through to today, the Ontario Ministry of Health, Long-Term Care Division provided $4.8 million in annual funding for community-based HPC initiatives that supported: (1) education initiatives for interdisciplinary community care providers and family physicians; (2) support and maintenance of hospice volunteer visiting programs; and (3) establishment of regional pain and symptom management teams. Funding for these initiatives is still provided annually [44]. Similarly, the provincial government in Alberta removed the per diem cost for hospices in 2004. One key informant described the impetus for this provincial funding initiative: "*They decided that the care you receive in the dying process should not be a financial burden to people. It sent a signal that hospice care was important and valued by government*."

An important funded policy intervention taken up by all provinces sampled was the implementation of HPC drug plans. These programs were significant interventions as they broadened the scope of the provision of HPC beyond what is allowed exclusively through the CHA (in that the CHA limits pharmaceutical coverage to hospital-based or physician-provided care). As explained by a key informant from British Columbia, the creation of the provincial 'Plan P' drug program in 2001 by the BC Ministry of Health provided medical equipment and pharmaceuticals to HPC patients outside of the hospital, including in hospice and home settings.

### 1c) Channels for Advocacy

Concerned citizens, advocacy organizations and individual local champions typically drove interventions designed to develop HPC. The work of these individuals and groups is of particular significance, as Canadians are generally known to have a death-denying culture discussed earlier, making it extremely difficult to advocate for change. One key informant explains how palliative care is regarded as not having as much influence as other health care issues: "*[HPC] just is not very sexy... not very attention getting*." Key informants identified numerous organizations and individuals that advocate for a stronger HPC system.

The Canadian Hospice Palliative Care Association (CHPCA), as a key national-level advocacy group, has perhaps been most instrumental in bringing attention to the need for developing HPC resources in Canada. Since its inception in 1991, the CHPCA has been acting as a national voice in public policy, awareness, and education for HPC care. Besides producing advocacy kits for HPC providers and interest groups, the CHPCA developed an instrumental document in 2002 entitled *A Model to Guide Hospice Palliative Care: Based on National Principles and Norms of Practice *[45]. One key informant explained that a landmark element of the model is its sensitivity to the needs of urban and rural communities alike:

...[the model] said that basically, every program should have some kind of inter-professional mechanism that people can come together and care plan. So, that doesn't mean that you have to live in urban Vancouver. You could have that inter-professional mechanism by telephone in anywhere in BC [British Columbia]. So [CHPCA was] trying to make that model that, in all ways...would be suitable for all care providers.

In addition to putting out instrumental documents, the CHPCA is the national association representing all provincial associations and over 500 hospice palliative care programs and services. In total, the CHPCA has a roster of more than 30,000 members including volunteers, researchers, home care program workers, health professionals, and other stakeholders. The mandate of the CHPCA includes: improving end-of-life care through collaboration; increasing awareness and knowledge about HPC to the public, health care providers and volunteers, and allocating resources and support services for caregivers.

The authors of key federal reports, namely The Honourable Sharon Carstairs and Dr. Frank Ferris, were frequently cited as advocates who used their influence to create initiatives to shift the existing HPC system. Similarly, within each province sampled, multiple champions were recognized for their instrumental role in progressing HPC on local, provincial and national levels. This group was comprised of community volunteers, palliative care specialists and researchers, and general health professionals, or those that became advocates after the death of a loved one.

Stakeholder organizations also introduced interventions at the provincial and national levels. For example, in Prince Edward Island, groups of concerned citizens advocated for increased public and government attention to HPC care after the release of the *Quality End-of-Life Care: The Right of Every Canadian *report [14]. Cancer organizations were also identified as instrumental advocacy groups in many of the provinces. For example, Cancer Care Ontario began to use their advocacy power to prioritize HPC by gathering data and disseminating information about care needs. Cancer agencies in British Columbia and Québec were also identified by key informants as significant contributors to designing interventions for HPC. However, as some key informants argued, the involvement of cancer societies can pose a limitation to embracing the intended breadth of HPC by associating such care too strongly with cancer care specifically.

### 2) Service Innovations

Alongside interventions, service innovations were identified as a primary circumvention strategy for HPC care. Essentially, service innovations are publicly- funded programs designed to work within the system to overcome existing limitations that HPC has inherited, such as: lack of specialist palliative care physicians, lack of funding, and geographical barriers (e.g., in remote communities). Service innovations were specific strategies and ideas designed to strengthen and support the ways in which HPC is delivered. Service innovations described herein are educational initiatives and HPC networks.

### 2a) Pallium

Pallium was an educational initiative funded through the federal Primary Health Care Transition Fund, which ran from 2004 to 2006 [46]. As a service innovation, Pallium focused on funding and developing innovative HPC resources and models, in education and training, for northern and western Canada [46]. Our key informants described Pallium as being a major success: "*...[Pallium] came and injected enthusiasm, money and expertise into the whole [HPC] field. I think that it gave a huge surge*." A variety of initiatives developed through Pallium funding. In Manitoba, annual HPC educational workshops were held in rural communities, with the assistance of travel funding from Pallium. Although Pallium ended in 2006, it provided a strong foundation for ongoing development and advancement of rural HPC.

### 2b) Educational Initiatives

A number of additional HPC education programs were identified by our key informants as playing a significant role in advancing HPC across Canada. The Comprehensive Advanced Palliative Care Education program, for example, was developed in Southwestern Ontario in 2007 by the Ministry of Health and Long Term Care to provide HPC education to primary care providers [43]. Specifically, the program offers an education curriculum facilitated by recognized local professionals with expertise in HPC care [43]. This program is recognized as a best practice model for Ontario and is being adopted across the province [43]. Similarly, in Ontario, the Palliative Care Integration Project facilitated the development of training modules and best practices for palliative care in the province's southeast region as a result of the release of the Ministry of Health and Long-Term Care's Report on the End-of-Life Care Strategy [47]. The Centre for Education and Research on Aging and Health at Lakehead University developed specialized curricula for palliative care education in long term care homes and First Nations communities. Similar HPC curriculums have been implemented across Canada within educational institutions.

As a result of an increased national focus on creating HPC services through the 1980s and 1990s, the Palliative Care Network of Quebec (PCNQ) established itself as a provincial champion of HPC education as well as advocacy [48]. The PCNQ acted as an agent of mobilization, bringing together interest groups and professionals to share their competencies [48]. Some educational initiatives have focused on developing research profiles along with educating the public in general. Also in Quebec, an early innovative HPC centre served later as a model for further development. La Maison Michel-Sarrazin, founded in 1985 through a subsidy from the Quebec Department of Health, provides HPC services, training for professionals, and conducts on-going research [49]. La Maison Michel-Sarrazin is also particularly important as it is the first francophone HPC program in the world, recognizing the needs of Canada's status as a bilingual nation. Similarly, the provincially-funded Hospice Project in British Columbia (1978) led to the development of the Victoria and Vancouver Hospices in 1980 and 1981 respectively, and later the Learning Centre for Palliative Care in 1999. These two hospices, as well as the Learning Centre for Palliative Care, have played pivotal roles in developing and strengthening HPC educational care resources and research.

### 2c) Innovative Health Service Delivery

Many of Ontario key informants emphasized the significance of the HPC regional networks as a major initiative that is advancing HPC in Ontario. In fact, these networks, together with the innovative shared care model which is being developed in one particular Local Health Integration Network, are being closely followed by other Canadian jurisdictions. As described by a key informant, HPC networks marry grassroots work together with broader systems support to provide a unique circumvention:

...on a system level we have never had a broad level of system support. So to see these networks forming is to see that there is a potential for policy changes to be made. And that the idea of hospice palliative care succeeding is to say that it can get out of the roots of the grass and into the tips of the blades, so that there are actually policies at an organizational level that also cross organizations in similar ways to help build a system that actually meets people's needs.

One specific HPC network, the Hamilton Niagara Haldimand Brant HPC Network, has endorsed a shared care model. Shared care is established when a specialist HPC team works in collaboration with front-line health providers, usually family physicians and home-care nurses [50]. This model of shared care promotes partnerships and collaboration between various practitioners, providing different levels of care to deliver seamless HPC to clients and their families in the home and other care settings. By using a shared care model, the Hamilton Niagara Haldimand Brant HPC Network aims to overcome the lack of HPC specialist training and utilize expertise across a continuum of health professionals in order to provide a more comprehensive care plan. Additionally, the shared care model aims to build capacity among health care professionals and informal caregivers [43].

In the same regard, Prince Edward Island and Nova Scotia collaborated in an inter-provincial project called the *Rural Palliative Homecare Project*, which was funded with monies from the Federal Health Transition in 1997. They received approximately one million dollars to initiate and evaluate their project, which formally came to an end in 2001. The objectives of the project were threefold. First, the rural palliative homecare model provided a framework to describe how to deliver HPC service to rural residents. Second, the model was used to guide the delivery of HPC services in three rural sites. Third, the project was evaluated in an effort not only to inform the structure of future HPC services in Nova Scotia and Prince Edward Island, but also to facilitate knowledge translation (i.e., to develop a model to be used in rural communities across Canada) [51]. This model was considered to have pioneered rural HPC services in Atlantic Canada and nationally, including the development of other education and evaluation tools [52]. After the conclusion of the rural palliative homecare strategy, Prince Edward Island appointed a provincial coordinator to assist its health regions to further implement integrated HPC [53]. Later, in 2005, Prince Edward *Island's Integrated Palliative Care Program *was recognized as national best practice [46].

### 3) New Alternative Structures

Particular forms of circumventions designed to overcome the many shortcomings in the HPC landscape are classified in this study as new 'alternative' structures because their focus lies outside the formal health care system. In particular, our data identified that independent hospice organizations are a strong circumvention to the limitations of the inherited system. Many regions across the country manage palliative care shortfalls by developing and maintaining independent volunteer hospices. There are various types of hospices supported by non-government resources including, for example, those that run residential facilities or day programs using primarily donated funds and provincial gaming revenues, and hospice societies that play a key role in advocacy and fundraising. Most of these programs meet unmet educational and service needs, effectively filling gaps left by the inheritances discussed earlier.

Telehealth, in particular, has been recognized for its ability to assist health care providers in gaining knowledge from outside sources (i.e. continuing education or specialist advice), which can minimize patients' need to travel for care [54]. Telehomecare strategies have been used across Canada to track or assist patients receiving HPC in their homes [25]. However, the provision of HPC homecare services through telemedicine requires proper infrastructure, especially in remote regions which may be lacking capacity to support technology [54]. Several HPC telehealth strategies have been implemented in the provision of HPC homecare to rural seniors, for example in rural Alberta and Prince Edward Island. In British Columbia, the Nurseline telehealth project, initially funded through Pallium, enables nurses to act as a first response to HPC questions from professionals and informal caregivers.

Similarly, the Canadian Virtual Hospice employs the use of the internet to relay information to providers, both informal and informal, and patients. The Canadian Virtual Hospice was created in 2001 through a joint initiative undertaken by HPC leaders across Canada. All of these alternative structures recognize that palliative care is not confined to certain hours of the day, and thus around the clock care be operationally made available.

Overall, our research results confirm that in order to overcome obstacles arising from inherited health systems and structures in Canada, the development of circumventions were essential for the progression of HPC. The exercise of circumventions has been recognized as being a complex practice, responding to the need to integrate practices, address geographical barriers, and respond to the complex/diverse needs of HPC patients and both formal and informal caregivers.

## Discussion

In Canada, we have inherited a death-denying culture of health care delivery characterized as being both highly curative and bio-medical in nature and, thereby, more interested in healing the bio-physical body rather than attending to the psycho-social and spiritual elements of the dying being [55]. Many of these system structures existed before HPC was formally developed in Canada and thus have intrinsically shaped the development of this form of care. These inheritances, as discussed include: (1) foundational health policies such as the CHA, together with national and provincial funding structures; (2) service structures and planning processes, such as the largely urban focused health system, and the speed at which health service progress takes place; and (3) health systems decisions such as regionalization. Similar to our concept of inheritances, Hutchison et al. argue that primary care developments in Canada have had to contend with three "policy legacies": (1) the federal/provincial division of power; (2) the structuring of private primary care practice via public payment, and; (3) the privileging of physician and hospital services [56]. The role of inheritances has also been discussed in the international literature. For example, a review of the development of HPC services in seven European countries noted the importance of existing health structures on program evolution [57].

As outlined above, a number of inherited structural elements have slowed the uptake of HPC care into the established systems of care. Given this, a number of initiatives have been implemented in an attempt to circumvent the established system of care. The exercise of circumventions is recognized as a complex practice, responding to the need to integrate practices, address geographical barriers, and respond to the complex/diverse needs of HPC patients and both formal and informal caregivers. The international literature has also noted geographical barriers to the development of HPC services. For example, the challenges of delivering HPC in rural and remote areas has been studied in Australia [58,59]. Clearly the circumventions have moved HPC services forward in spite of the identified inheritances. Further, many of the circumventions identified by the key informants are clearly here to stay as they are now embedded in the system. For example, HPC drug plans will be very difficult to eradicate, as will Canada's Compassionate Care Benefit. Both these circumventions are responses to Carstairs', Kirby's and Romanow's landmark reports calling for significant health system restructuring, including HPC improvements. For example, Romanow recommended that the CHA be revised to include coverage for home care services specific to HPC. In addition, he recommended that the federal government provide more support to informal caregivers [25].

### Ways Ahead in the Evolution of HPC

There is no question that advocacy will continue to advance HPC across the country. Concerned citizens, advocacy organizations and individual local champions will contribute to, if not drive, change. They will continue to support new alternative systems, service innovations and initiatives to shift the system. Advocacy organizations such as CHPCA are central to the ongoing development of HPC in Canada and exist as a foundational governance structure for ongoing progress. The work of CHPCA and the many provincial HPC associations, together with citizen advocacy, has the potential to assist with minimizing the cultural and social taboos around death and dying prevalent in both the Canadian psyche and the Canadian health care system. One key informant explains:

They're [decision-makers] not people who actually want to give any attention to this [HPC]. It isn't something people want to talk about. So, it really does take some exposure of people who are in decision-making roles to [know] how awful it can be if somebody dies badly [in order to prioritize HPC]. And I think, you know, that's a terrible thing and I don't wish that on anybody, but I do see how naïveté about what this [HPC] is about gets in the way of good decision-making when people are just thinking life's wonderful and we're all going to live forever.

Advocacy may also further shift the HPC mentality away from a 'curative mindset' and towards a 'caring mindset' where a person, as a whole being, is considered rather than solely the bio-physical body.

A number of key informants are hopeful that HPC can be more seamlessly integrated into Canada's primary health care system in a comprehensive manner. One key informant suggested how that might take place:

I think there needs to be a task group that sits down and starts to put all this together in some comprehensive fashion -- much as we do for birthing. I mean, we have neonatal intensive care units of varying degrees. We fund those. There isn't a single palliative care unit in Ontario that is funded with specific Ministry of Health dollars. There are residential hospices that are funded but they can only offer a limited amount of care and that's where the Ministry seems to have put dollars, which is not going to affect most of the people who die who need any kind of in-patient care.

Continued movement along the lines of the shared care teams, where HPC is provided via a primary health care model, has the potential to assist with this desired integration. If this were to occur, HPC services would have to be considered in relation to primary health care indicators, where concerns around access and seamless transitions, for example, can be tracked and evaluated over time.

### Knowledge Gaps

The current state of HPC in Canada could improve substantially if evaluation were to be prioritized. Our review of the grey literatures revealed very little formal evaluation. While we are beginning to see improvements in evaluation efforts for health services [51,60], there remains a dearth of evaluative research on HPC service delivery in Canada, thus resulting in a lack of data and, consequently, lack of traceability and evidence specific to best practices. This is particularly needed with rural geographies of Canada where timely access and seamless transitions to care are problematic given the medically underserviced nature of these often distant and remote areas [25]. The same is needed with vulnerable populations, such as First Nations and Inuit peoples of Canada who have significantly lower life expectancies and a much higher incidence of chronic illness when compared to the general Canadian population [61-63].

### Limitations

There are a number of limitations in the research presented in this paper. Limitations involving the interviews are explored first. Some of the interviews were conducted in French and translated into English, increasing the potential for differences in interpretation due to translation. Additionally, as interviews were conducted by multiple interviewers across the provinces, issues may not have been probed consistently. As only some provinces were included in this study, the state of HPC in every province and territory in Canada was not captured.

Much as the CHPCA characterizes HPC as a patchwork quilt, so too is the state of documentation regarding HPC service and delivery across Canada. As a result, this research is bounded by the availability of information and the cumulative knowledge of the research team. Grey literature is difficult to search systematically so there is the potential that key reports or various other forms of information may have been missed. Given the complexity of HPC, what is presented here is a first attempt to make sense of the breadth of the evolution of HPC in Canada.

We mitigated these limitations by having frequent research meetings to discuss findings, and by fact checking as much as possible; this employed the use of the internet, the scholarly literature, and expert researchers. Furthermore, this involved tracking and reviewing the first journals of palliative care in Canada as well as and having informal conversations with pioneers in the field, and stakeholders representing key organizations.

## Conclusions

The purpose of this research study was to analyze the evolution of HPC in Canada through a qualitative comparative case study of seven provinces. Forty-two key informants were interviewed to collaborate in the development of timelines tracking the evolution of HPC. Additional data came from scholarly and grey literature searches. The results of this study revealed a number of inherited structural elements that have prevented the uptake of HPC care into the established systems of care including foundational health policies, service structures and planning and health system decisions. Various circumventions have developed in response to these inheritances, including: interventions to shift the system, service innovations, and new alternative structures. As discussed in our framework of inheritances, health services are typically slow to adapt to change, thus limiting the possibility of developing new strategies that are immediately responsive to population needs, including in HPC care. This particular issue was emphasized by a key informant who discussed measuring HPC evolution in decades rather than years:

I measure palliative care in decades...but some of the real difficulties in advocacy and building and kind of bringing hospice palliative care forward has certainly been such, and continues to be, that you really can't measure much growth in terms of months or years. Canada remains a 'work in progress'.

However, examination of HPC progression and activity at the provincial and regional levels demonstrates that while evolution has been slow, it has actually been continuous in responding and adapting to the changing needs and expectations of Canadians. This is particularly true in the face of the anticipated growth in demand in the coming decade as Canada's population continues to rapidly age.

Good health service planning requires us to examine past events in order to determine what has worked and has failed before moving on to the future. As evidenced in the research presented herein, we must recognize and build on the successes of individuals and organizations to enhance HPC delivery through circumventions developed in light of foundational inheritances. As demonstrated within this research paper, HPC care remains at the margins of the health care system. Our study confirms that many of the innovative initiatives discussed would not have been possible without federal assistance and provincial direction, but ultimately it is the ingenuity and perseverance of individuals and organizations with a compassion for HPC that essentially moves HPC forward.

Certainly, one of many ways forward is to focus on entrenching some of the circumventions into inheritances in order to have them be sustainable in the long term. For example, having residential hospice programs securely funded by Canadian tax dollars will ensure their ongoing existence. Undoubtedly, as current circumventions such as these become inheritances, new circumventions will develop and, in doing so, continue the progress being made in HPC throughout Canada.

## List of Abbreviations

CHA: Canadian Health Act; CHPCA: Canadian Hospice Palliative Care Association; CHT: Canadian Health Transfer; CTA: Canadian Transfer Act; HPC: Hospice Palliative Care; RHA: Regional Health Authority

## Competing interests

The authors declare that they have no competing interests.

## Authors' contributions

All authors with the exception of SD and LD oversaw data collection and management for specific provinces and attended a team data analysis meeting where the manuscript outline was jointly prepared. AW led preparing the manuscript draft, with some sections being contributed by VAC, LD, and SD. All authors reviewed and edited the manuscript. All authors have approved the submission version.

## Pre-publication history

The pre-publication history for this paper can be accessed here:

http://www.biomedcentral.com/1472-6963/10/147/prepub
